# Increased blood immune regulatory cells in severe COVID-19 with autoantibodies to type I interferons

**DOI:** 10.1038/s41598-023-43675-w

**Published:** 2023-10-13

**Authors:** Fatemeh Saheb Sharif-Askari, Narjes Saheb Sharif-Askari, Shirin Hafezi, Hawra Ali Hussain Alsayed, Balachandar Selvakumar, Mariam Wed Abdelaziz Eladham, Bushra Mdkhana, Ola Salam Bayram, Mohamad-Hani Temsah, Rabih Halwani

**Affiliations:** 1 Research Institute for Medical and Health Science, University of Sharjah, Sharjah, UAE; 2 Department of Pharmacy Practice and Pharmacotherapeutics, College of Pharmacy, University of Sharjah, Sharjah, UAE; 3 Department of Clinical Sciences, College of Medicine, University of Sharjah, Sharjah, UAE; 4 Department of Pharmacy, Rashid Hospital, Dubai Academic Health Corporation, Dubai, UAE; 5 Department of Pediatrics, College of Medicine, King Saud University, Riyadh, Saudi Arabia; 6 Immunology Research Lab, College of Medicine, King Saud University, Riyadh, Saudi Arabia

**Keywords:** Immunology, Autoimmunity

## Abstract

The hallmark of severe COVID-19 is an uncontrolled inflammatory response, resulting from poorly understood immunological dysfunction. While regulatory T (Treg) and B (Breg) cells, as the main elements of immune homeostasis, contribute to the control of hyperinflammation during COVID-19 infection, we hypothesized change in their levels in relation to disease severity and the presence of autoantibodies (auto-Abs) to type I IFNs. Cytometric analysis of blood of 62 COVID-19 patients with different severities revealed an increased proportion of conventional (cTreg; CD25^+^FoxP3^+^) and unconventional (uTreg; CD25^-^FoxP3^+^) Tregs, as well as the LAG3^+^ immune suppressive form of cTreg/uTreg, in the blood of severe COVID-19 cases compared to the milder, non-hospitalized cases. The increase in blood levels of cTreg/uTreg, but not LAG3^+^ cTreg/uTreg subtypes, was even higher among patients with severe COVID-19 and auto-Abs to type I IFNs. Regarding Bregs, compared to the milder, non-hospitalized cases, the proportion of IL-35^+^ and IL-10^+^ Bregs was elevated in the blood of severe COVID-19 patients, and to a higher extent in those with auto-Abs to type I IFNs. Moreover, blood levels of cTreg, LAG3^+^ cTreg/uTreg, and IL-35^+^ and IL-10^+^ Breg subtypes were associated with lower blood levels of proinflammatory cytokines such as IL-6, IL-17, TNFα, and IL-1β. Interestingly, patients who were treated with either tocilizumab and/or a high dose of Vitamin D had higher blood levels of these regulatory cells and better control of the proinflammatory cytokines. These observations suggest that perturbations in the levels of immunomodulatory Tregs and Bregs occur in COVID-19, especially in the presence of auto-Abs to type I IFNs.

## Introduction

COVID-19, resulting from SARS-CoV-2 infection, is still a global health challenge. Although most patients present with subclinical disease and a mild form of illness, about one-fifth develop severe illness that requires hospitalization and oxygen support. The clinical and pathological investigation of patients with severe COVID-19 has revealed that the severity of the disease is associated with a number of factors, including specific patient characteristics, such as obesity, diabetes, and old age^1^. Additionally, immunopathologic abnormalities in the lung tissue of these patients correlate with the onset and progression of the disease^1,2^.

To combat the immunopathology of viral pneumonia, several studies have shown that immune regulatory cells play crucial roles in controlling viral pneumonia and viral-induced acute lung injury^3–5^. Regulatory cells inhibit multiple immune cells, such as CD8^+^, CD4^+^ T cells, monocytes, NK cells, as well as B cells, thus regulating unfavorable immune responses during different stages of infection and disease transition from mild to severe^6^.

Regulatory T cells (Tregs) expressing the transcription factor Foxp3 are considered central to restoring immune homeostasis during infection and preventing immune overactivation. In a mouse model study of infection with respiratory syncytial virus, Foxp3^+^ Tregs were shown to significantly accumulate in alveolar spaces and lower immunopathology of the lung by modulating the CD8^+^ T cell response during infection^5^. Another study of acute lung injury induced by lipopolysaccharide (LPS) demonstrated the accumulation of Foxp3^+^ Tregs in lung alveoli and their essential role in alleviating acute lung injury in mice^3^.

Moreover, regulatory B cells (Bregs) suppress immune cell activation and enhance the conversion of T cells to Tregs through the production of anti-inflammatory mediators like IL-10 and IL-35, which in turn, attenuate immune responses^7^. Additionally, Bregs have been shown to be recruited to the site of infection to suppress damaging inflammation in the lung tissues through the production of IL‐10 or IL-35^8^.

Recently, the role of auto-Abs (autoantibodies) neutralizing type I IFNs among patients with severe COVID-19 was revealed. These auto-Abs are common in the general population, and their prevalence increases after 65 years^9^. Moreover, one-fifth of fatal COVID-19 cases were associated with preexisting auto-Abs neutralizing type I IFNs^9,10^. An experimental study has also shown a link between LAG3 Tregs and type I IFN signaling by demonstrating that blocking the receptor for type I IFNs (IFNAR1) by an antibody to IFNAR1 significantly reduced the proliferation of LAG3 Tregs and their immunosuppression effect in mice injected with myeloma cells^11^.

In light of these reports, we hypothesized that Tregs and Bregs might contribute to the control of systemic inflammatory features of severe disease, especially in patients who have preexisting neutralizing auto-Abs to type I IFNs. Hence, we determined the level of circulating Tregs and Bregs in a cohort of confirmed COVID-19 patients with and without neutralizing auto-Abs to type I IFNs and of varying severity.

## Methods

### Patients and clinical evaluation

We included 62 adult patients aged 18 years and older who were diagnosed with COVID-19-related pneumonia and confirmed to be infected with SARS-CoV-2 by PCR at Rashid Hospital in Dubai between September 2020, and January 2021. Patients younger than 18 years old, pregnant women, and patients with active malignancies were excluded from the study. Among 62 patients, 38 were diagnosed with severe COVID-19, requiring hospitalization and intensive care unit stay. The severity status of COVID-19 was defined as pneumonia that required high-flow oxygen therapy or non-invasive ventilation^12^. Blood samples were taken from the patient as part of routine clinical care, as well as from 18 healthy donors to serve as controls. Neither the patients nor the healthy controls had been vaccinated for COVID-19 at the time of blood collection. Table 1 presents clinical and laboratory data at admission.

**Table 1:** Baseline clinical characteristics of the study population.

Variables	Healthy controls (n = 18)	Non-hospitalized asymptomatic patients (n = 24)	Hospitalized patients (n = 38)		P-value*
			auto-Abs IFNs^+^ (n = 20)	auto-Abs IFNs^−^ (n = 18)	
Age, years	38 (30–49)	37 (30–48)	55 (47–68)	53 (36–69)	0.739
Male sex	13 (72)	14 (61)	18 (90)	16 (89)	0.911
BMI	23 (22–24)	24 (22–27)	28 (22–35)	26 (23–30)	0.114
DM	–	2 (10)	12 (63)	7 (41)	0.316
Hypertension	–	4 (17)	3 (15)	3 (17)	0.888
Serum markers of COVID-19 severity (normal range)	–	–			
C-reactive protein (1.0–3.0 mg/L)	–	–	57 (30–246)	19 (8–104)	0.055
D-Dimer (0–0.5 μ/mL)	–	–	1.7 (0.9–2.9)	1.3 (1.2–2.2)	0.552
Ferritin (10–204 ng/mL)	-	–	789 (292–1343)	712 (192–1290)	0.652
Pro-inflammatory cytokines					
IL-6	–	–	136 (66–506)	244 (46–602)	0.710
TNFα	–	–	267 (144–358)	171 (105–626)	0.781
IL-17	–	–	358 (51–628)	86 (34–115)	0.015
IL-1β	–	–	69 (28–111)	53 (12–95)	0.554
COVID-19 supportive medication	–	–			
Vitamin D	–	–	11 (55)	8 (44)	0.516
Tocilizumab	–	–	10 (50)	6 (33)	0.299
Clinical outcomes	–	–			
Mechanical ventilation	–	–	12 (60)	10 (56)	0.782
Death	–	–	17 (85)	8 (44)	0.016

Data are n (%) or median (IQR).

*BMI* body mass index, *CRP* C-reactive protein, *DM* diabetes mellitus, *VitD* vitamin D.

*The p-values for different variables (age = 0.002, male sex = 0.072, BMI = 0.114, DM = 0.001, and hypertension = 0.340) were calculated to compare healthy controls, non-hospitalized asymptomatic patients, hospitalized patients with autoantibodies to type I IFNs, and those without autoantibodies to type I IFNs.

This study was approved by the Dubai Scientific Research Ethics Committee (DSREC), Dubai Health Authority at Rashid Hospital (DSREC-12/2020_02). All participants provided written informed consent. All methods were performed in accordance with the relevant guidelines (Declaration of Helsinki and the Belmont Report) and regulations (DSREC rules).

### Cell preparation and cytometry analysis

Isolation of peripheral blood mononuclear cells (PBMCs) was performed using a Ficoll gradient (Axis-Shield, Norway). The unstimulated PBMCs were stained with APC anti-human CD19 antibody (BioLegend cat #302212), APC/Cyanine7 anti-human CD138 antibody (BioLegend cat #356528), PerCP/Cyanine5.5 anti-human CD1d antibody (BioLegend cat #350312), APC anti-human CD25 antibody (BioLegend cat #302610), and PE anti-human CD223 (LAG-3) antibody (BioLegend cat #369306). Following surface staining, cells were stained with PE anti-human EBi3 antibody (BioLegend cat #360904), PE/Cy7 anti-human IL-10 antibody (BioLegend cat #501420), and FITC anti-human FOXP3 antibody (BioLegend cat #320106). For each antibody, the corresponding isotype controls were used. All cells were analyzed with BD LSR II flow cytometer using DIVA software version 8.0.

### Detection of auto-Abs neutralizing type I IFN

Plasma samples from 38 severe COVID-19 patients were isolated from the blood via standard centrifugation. ELISA was performed as described previously^9^. Briefly, 96-well ELISA plates were coated by human IFN-α2 recombinant protein (2 μg/ml; Miltenyi Biotec, Bergisch Gladbach, Germany; catalog no. 130-093-874;) and human IFN-ω recombinant protein (eBioscience, CA, USA; catalog no. BMS304) and incubated overnight at 4 °C. Coated plates were then washed with phosphate-buffered saline with 0.005% Tween® 20 detergent (TBST), blocked by incubation with 5% nonfat dry milk in TBST, washed with TBST, and incubated with 1:250 dilutions of plasma from the patients for 2 h at room temperature. Horseradish peroxidase (HRP)-labeled anti-human IgG, IgM, or IgA (Abcam, Cambridge, MA, USA; catalog no. ab102420) were added to a final concentration of 2 μg/ml. To develop the plate, 100 μl/well of TMB substrate solution (TMB Substrate Kit, Thermo Fisher Scientific, MA, USA; catalog no. 34021) was added and allowed to stand for 5 min. The reaction was stopped with 2 M sulfuric acid (100 μl/well) and optical density at 450/630 nm was measured via a BioTek microplate reader.

### Cytokine levels in plasma of COVID-19 patients

Plasma levels of IL-35, IL-10, IL-6, IL-17, TNFα, and IL-1β cytokines concentrations in 62 COVID-19 patients and 18 healthy controls were determined using commercially available ELISA kits (Human IL-35, NBP3-06774, Novus Biologicals; human IL-10, ab100549, Abcam; human IL-6, DY206-05, R&D; human IL-17, DY317-05, R&D; human TNFα, ab181421, Abcam; human IL-1b, DY201-05, R&D). Assays were performed strictly following the manufacturer’s instructions. All samples were measured in duplicate.

### Statistical analysis

In the unadjusted univariate analysis, for continuous variables, means and SDs or medians and interquartile ranges were reported, as appropriate. Comparison between two independent groups was performed using the Student’s t-test or Mann–Whitney test, depending on the skewness of data. For comparisons involving three or more than three groups, a one-way ANOVA was used. Additionally, linear regression analysis was conducted, with adjustments made for patients' age and diabetes mellitus (DM). For categorical variables, percentages were compared across the groups using a χ^2^ test (Chi-square test analyses). For the survival analysis, the presence of auto-Abs neutralizing type I IFNs with mortality was evaluated by a Cox proportional hazards regression model adjusted for patients’ demographic factors (age, gender, and body mass index), comorbidities (DM), and COVID-19-related severity serum markers (D-dimer and C-reactive protein). Kaplan–Meier survival curves were then constructed to show cumulative survival over the 29-day period. Furthermore, we determined to have at least 18 samples per group to achieve a significant power value. We assumed a Cohen's d value of 1.12, a power of 0.9, a significance level of 0.05, and a two-sided test^13,14^. Analysis was performed using R software (v 3.0.2), SPSS Version 26 (IBM Corporation, Chicago, USA), and GraphPad Prism 8 (GraphPad Software Inc., San Diego, USA). All tests were two-tailed and a P value of less than 0.05 was considered statistically significant.

### Ethics approval

This study was approved by the Dubai Scientific Research Ethics Committee (DSREC), Dubai Health Authority at Rashid Hospital (DSREC-12/2020_02).

### Consent to participate

All participants provided written informed consent.

## Results

### Patient’s cohort

A total of 24 asymptomatic non-hospitalized COVID-19 patients and 38 hospitalized patients with severe COVID-19 were recruited. Around half of the severe COVID-19 (n = 20, 53%) cases were positive for auto-Abs against type I IFNs. All of the patients with severe COVID-19 were admitted to the intensive care unit (ICU), and blood was sampled during the cytokine storm period. The clinical characteristics of patients are detailed in Table 1.

### Upregulation of peripheral blood Tregs in patients with severe COVID-19, and to a greater extent in those with neutralizing auto-Abs to type I IFNs

There remains considerable controversy regarding the level of Tregs in COVID-19^15,16^. We, therefore, decided to determine how the different subsets of Treg correlate with COVID-19 severity and how this level associates with the presence of auto-Abs to type I IFNs.

**Figure 1:**
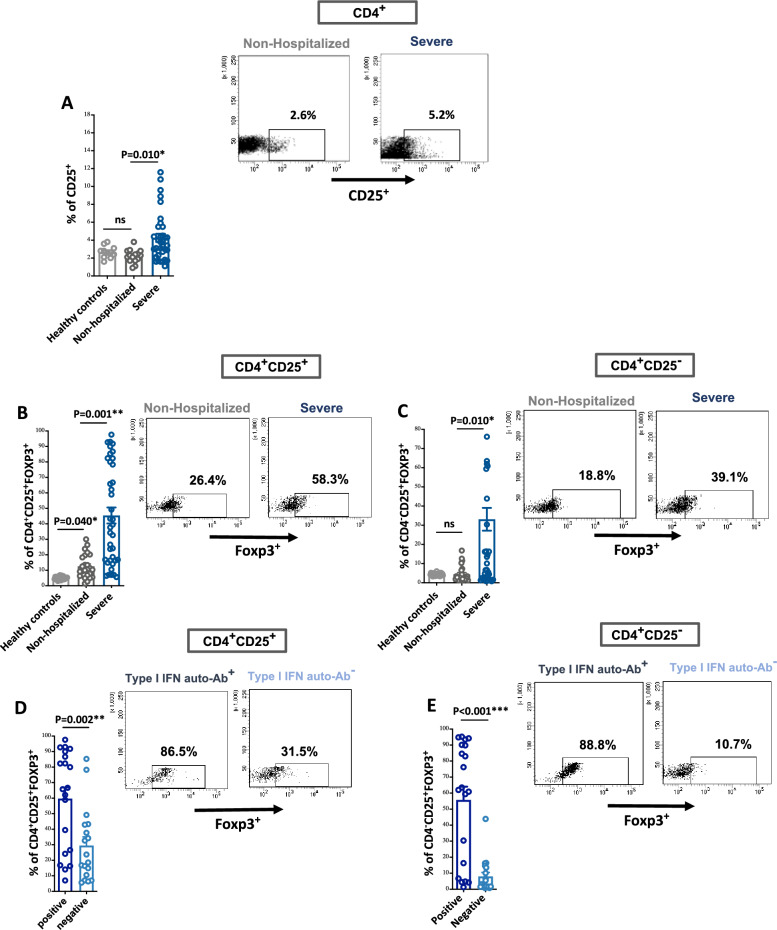

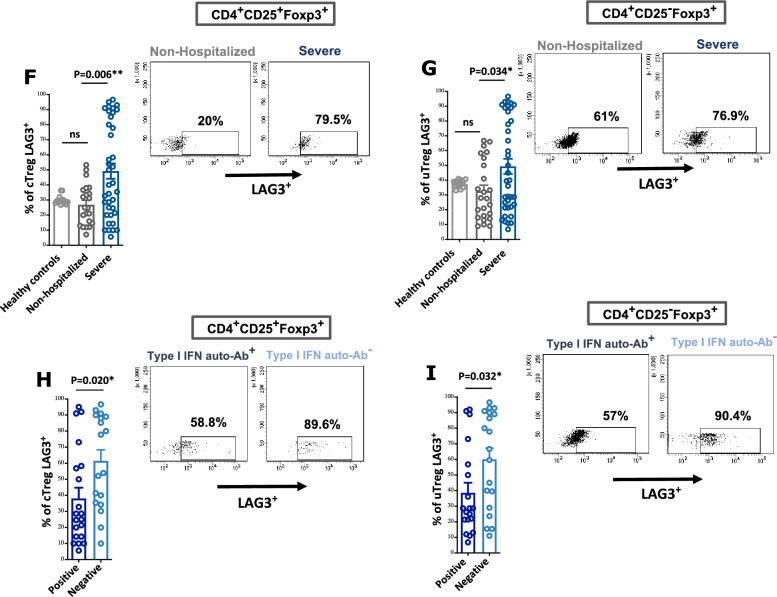
Levels of conventional and unconventional Tregs in the PBMCs of patients with COVID-19 with and without auto-Abs to type I IFNs. (**A**) Levels of CD4^+^CD25^+^ cells in the PBMCs of patients with severe COVID-19 and non-hospitalized patients. (**B–E**) Levels of CD25^+^Foxp3^+^ Tregs (cTreg) and CD25^-^Foxp3^+^ Tregs (uTreg) in the PBMCs of patients with severe COVID-19, both with and without auto-Abs to type I IFNs, and those who were non-hospitalized. (**F–I**) Expression levels of CD25^+^Foxp3^+^LAG3^+^ (LAG3^+^ cTreg) and CD25^-^Foxp3^+^LAG3^+^ (LAG3^+^ uTreg) cells in the PBMCs of patients with severe COVID-19, both with and without auto-Abs to type I IFNs, and those who were non-hospitalized. Events were recorded and analysed by using BD FACSDiva version 8.0. Statistical tests; Comparison between patients with and without auto-Abs to type I IFNs was performed using unpaired t-test or Mann–Whitney U test, depending on the skewness of the data. Linear regression models adjusted for patient's age and DM was utilized for other analysis. P value of < 0.05 was considered significant.

For that, we evaluated blood levels of CD25^+^_,_ conventional Tregs (cTreg; CD25^+^Foxp3^+^), and unconventional Tregs (uTreg; CD25^-^Foxp3^+^) in COVID-19 patients with different severity. Of note, we found higher levels of CD25^+^ as well as cTregs/uTregs in the blood of patients with severe COVID-19 compared to those of milder cases (around 33% increase in frequency of cTregs/uTregs, P = 0.001; Fig. 1A–C). Moreover, among patients with severe COVID-19, the levels of cTregs/uTregs were even higher in the blood of patients with auto-Abs to type I IFNs compared to their counterparts (30% and 48% increase in frequency of cTregs and uTregs, respectively, P = 0.002; Fig. 1D,E).

LAG3^+^ cTregs and LAG3^+^ uTregs are considered Treg subsets possessing the ability to produce a large amount of IL-10 and regulate immune responses in different experimental and clinical settings^17–21^. To determine if these aberrant Tregs in patients with severe COVID-19 associate with an immune suppressive capability, we measured the level of suppressive effector molecule LAG3^+^ within cTregs/uTregs. A Higher level of LAG3 in cTregs/uTregs associated with more severe COVID-19 disease (around 20% increase in the frequency of LAG3^+^ cTregs/uTregs, P = 0.03; Fig. 1F,G). However, we noticed that in severe COVID-19 patients with auto-Abs to type I IFNs, cTregs/uTregs had lower level of LAG3^+^ suppressive effector molecule compared to their counterparts (22% decrease in the frequency of LAG3^+^ cTregs/uTregs, P = 0.03; Fig. 1H,I), which may suggest a lower immunoregulatory capability of cTregs/uTregs from auto-Abs IFNs positive patients. However, confirming that requires more functional studies in a larger cohort of patients.

### Upregulation of peripheral blood IL-35^+^IL-10^+^ Bregs in patients with severe COVID-19, and to a greater extent in those with neutralizing auto-Abs to type I IFNs

Bregs are known to suppress immune responses through the secretion of IL-10 and IL-35^18^. However, little is known about IL-35 and IL-10 producing Bregs in the context of COVID-19. We assessed blood levels of Bregs expressing IL-35 and IL-10 surface markers in patients with different severities of COVID-19. The ability of Breg to produce these cytokines has been previously reported to contribute to Breg-mediated immune suppression mechanisms in rheumatoid arthritis patients^18^.

**Figure 2:**
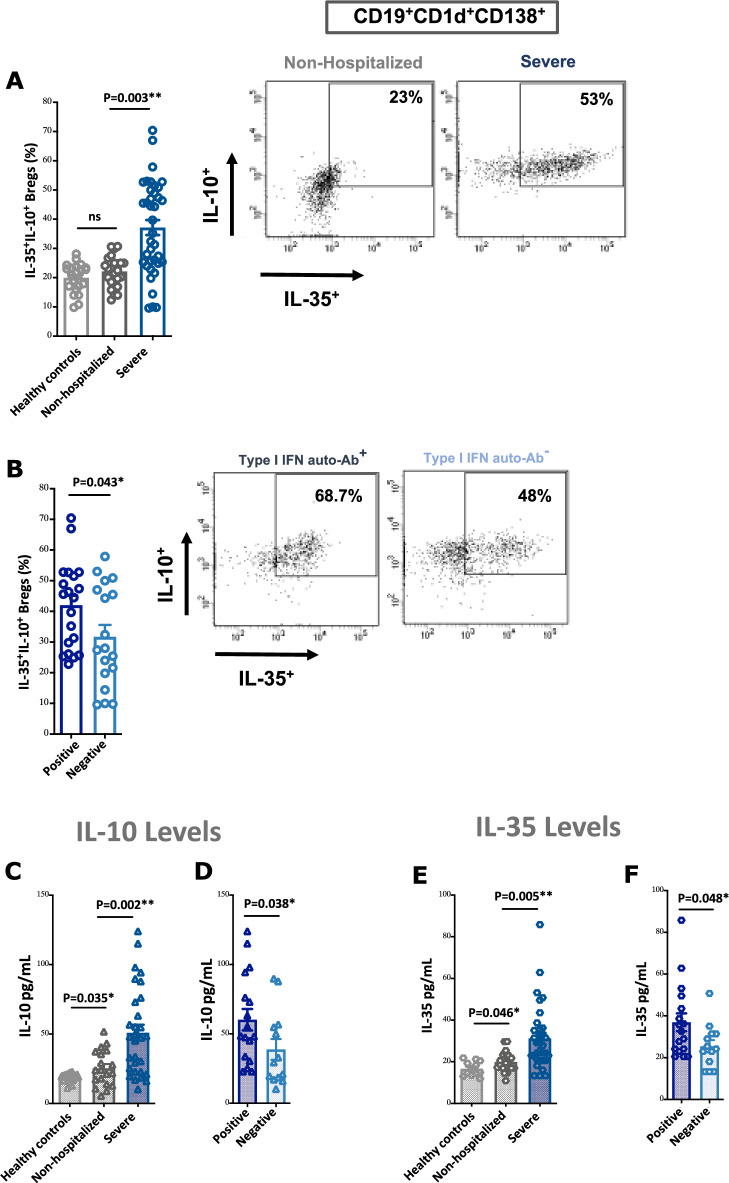
Levels of regulatory B cells in the PBMCs of patients with COVID-19 with COVID-19 with and without auto-Abs to type I IFNs. (**A,B**) Levels of IL-35 and IL-10 producing CD138^+^ CD1d^+^ Bregs in the PBMCs of patients with severe COVID-19, both with and without auto-Abs to type I IFNs, and those who were non-hospitalized. (**C–F**) Levels of IL-10 and IL-35 in plasma of patients with severe COVID-19, both with and without auto-Abs to type I IFNs, and those who were non-hospitalized. Events were recorded and analysed by using BD FACSDiva version 8.0. Statistical tests; Comparison between patients with and without auto-Abs to type I IFNs was performed using unpaired t-test or Mann–Whitney U test, depending on the skewness of the data. Linear regression models adjusted for patient’s age and DM was utilized for other analysis. P value of < 0.05 was considered significant.

Our results revealed an increase in the level of IL-35^+^IL-10^+^ producing CD138^+^CD1d^+^ Bregs (IL-35^+^IL-10^+^ Bregs) in the blood of severe patients compared to non-hospitalized patients with COVID-19 (30% increase in the frequency of IL-35^+^IL-10^+^ Bregs, P = 0.003; Fig. 2A). Notably, among patients with severe COVID-19, the level was even higher in the blood of patients with auto-Abs to type I IFNs compared to their counterparts (20% increase in the frequency of IL-35^+^IL-10^+^ Bregs, P = 0.04; Fig. 2B). The blood levels of IL-35 and IL-10 immunosuppressive cytokines followed the same increasing trend (Fig. 2C–F).

### Breg and Treg upregulation is associated with lower levels of COVID-19 proinflammatory cytokines in the blood

Furthermore, to assess the immunoregulatory effects of cTregs/uTregs, LAG3^+^cTregs/uTregs, and IL-35^+^IL-10^+^ Bregs on COVID-19 induced immune activation, we correlated the levels with serum markers of COVID-19 severity such as CRP and ferritin as well as with different circulatory proinflammatory cytokines such as IL-6, IL-17, TNFα, and IL-1β.

**Figure 3:**
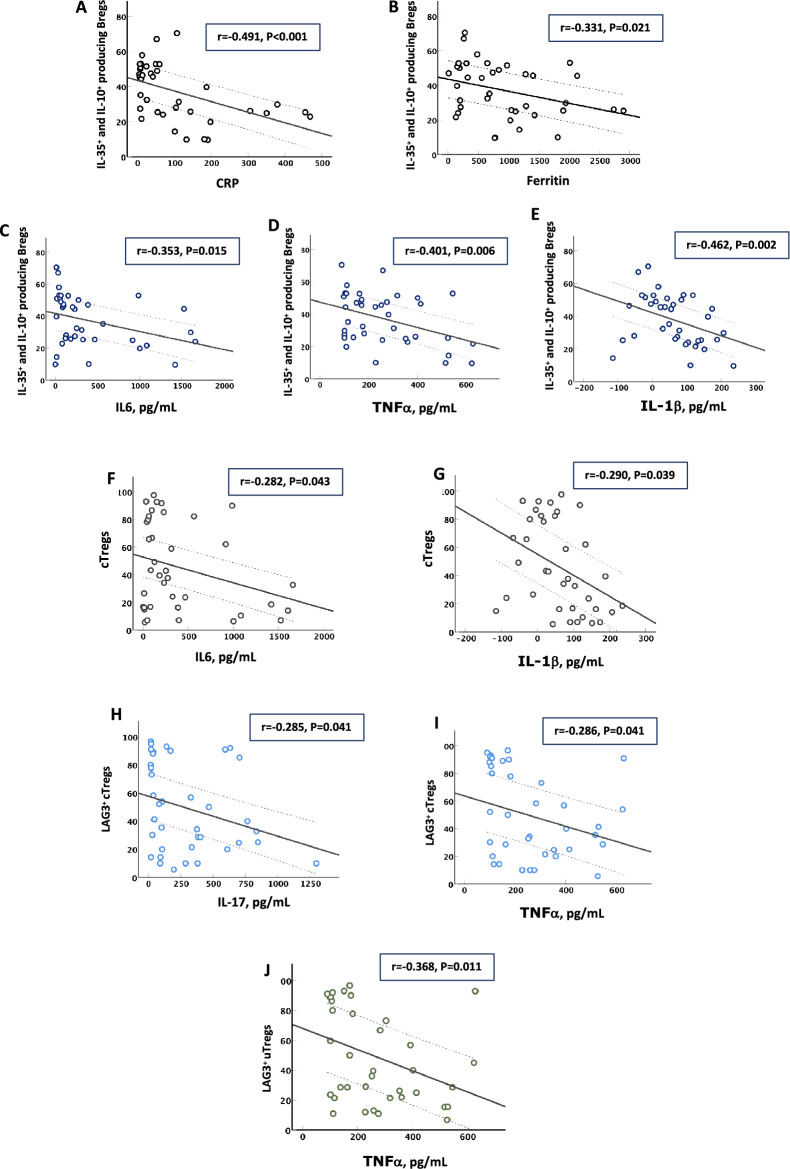
COVID-19 patients with higher levels of Tregs and Bregs in the PBMCs have lower levels of proinflammatory cytokines in the plasma. (**A–E**) Correlation between levels of IL-35 and IL-10 producing CD138^+^ CD1d^+^ Bregs and serum markers of COVID-19 severity such as CRP, D-Dimer, ferritin, and proinflammatory cytokines such as IL-6, IL-17, TNFα, and IL-1β. (**F,G**) Correlation between levels of cTreg and IL-6 and IL-1β. (**H–J**) Correlation between levels of LAG3 cTreg/uTreg and IL-17 and TNFα. Statistical test: Pearson’s correlation or Spearman’s coefficient was used based on the normality of data, with a two-sided test for significance (P < 0.05, considered significant).

Interestingly, our results showed that blood levels of IL-35^+^IL-10^+^ Bregs total Tregs, and LAG3^+^cTregs/uTregs subsets were associated with lower serum levels of CRP and ferritin, as well as reduced blood levels of proinflammatory cytokines (Fig. 3A–J, Supplementary Fig. S1).

### Tocilizumab and VitD enhance the blood levels of Bregs and Tregs

**Figure 4:**
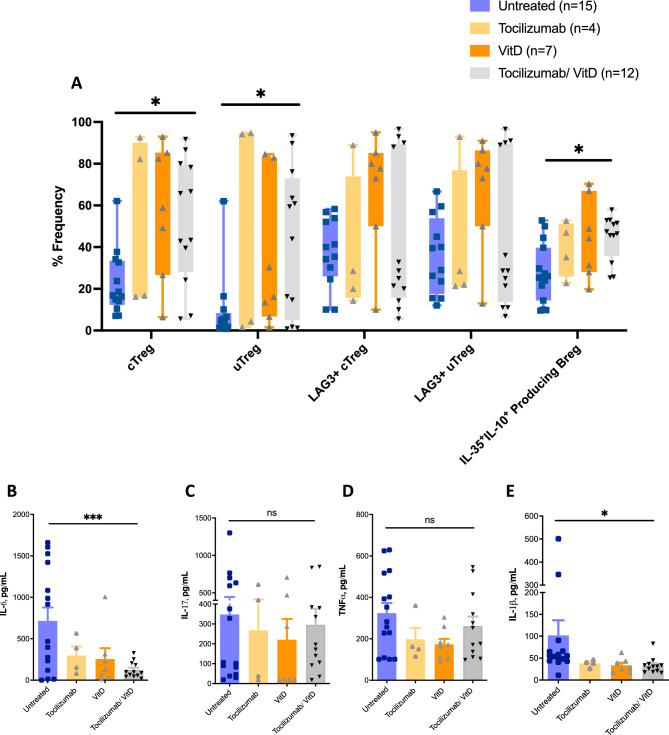
Tocilizumab and Vitamin D enhances the blood levels of Bregs and Tregs. (**A**) Levels of cTreg/uTreg, LAG3^+^ cTreg/uTreg, and IL-35 and IL-10 producing CD138^+^ CD1d^+^ Bregs in the PBMCs of tocilizumab and/or Vitamin D (VitD) treated patients with severe COVID-19. (**B–E**) Levels of IL-6, IL-17, TNFα, IL-1β in the PBMCs of tocilizumab and/or VitD treated patients with severe COVID-19. Tocilizumab was administered as a one-time treatment to patients. Statistical test: comparison was done using a one-way ANOVA test. *ns* non-significant. *P < 0.05, **P < 0.01, ***P < 0.001, ****P < 0.0001.

Next, we were interested in evaluating the effect of COVID-19 related medications on the blood levels of IL-35^+^IL-10^+^ Bregs as well as cTregs/uTregs subsets and LAG3^+^cTregs/uTregs. Tocilizumab and/or high dose of VitD significantly elevated blood levels of IL-35^+^IL-10^+^ Bregs as well as cTregs/uTregs subsets of treated patients compared to untreated patients (Fig. 4A). Accordingly, upregulation of these regulatory immune subtypes was associated with lower levels of proinflammatory cytokines (Fig. 4B–E), in particular IL-6 and IL-1β, in the blood of treated severe COVID-19 patients (Fig. 4B,E).

## Discussion

This study reports an increase in levels of both conventional and unconventional subtypes of Foxp3^+^ Tregs (cTregs/uTregs), as well as IL-35^+^ and IL-10^+^ producing Bregs, in the blood of COVID-19 patients during infection and relative to disease severity. The findings align with previous reports that have correlated higher blood levels of different subtypes of Treg including Foxp3^+^ Tregs with the severity of COVID-19^15,22–26^. Notably, the frequencies of Foxp3^+^ Tregs were shown to increase from day 7 to day 21 post-symptom onset in the blood of severe cases but not in mild cases^25^. Moreover, an increased gene expression level of LAG3 was detected in peripheral blood Foxp3^+^ Tregs^15^ and in lung autopsies of severely ill COVID-19 patients^27^. In our study, LAG3^+^ Tregs correlated with a lower level of serum proinflammatory cytokines in COVID-19 patients, suggesting that immune suppressive LAG3^+^ Tregs might play a key role in controlling the cytokine storm associated with severe disease.

Moreover, while the levels of cTregs/uTregs subsets were strikingly higher in the blood of severe patients with positive auto-Abs to type I IFNs compared to their counterparts, the level of LAG3^+^ cTregs/uTregs was considerably lower among these patients. The reason could be that sustained Type I IFN signaling is key for the expansion of LAG3^+^ Tregs, as it was shown that blocking type I IFNs receptor by IFNAR1 antibody hindered myeloma-driven LAG3^+^ Treg expansion^11^. The observed low LAG3 expression in cTregs/uTregs of patients with positive auto-Abs to type I IFNs could reduce the ability of these Tregs to control cytokine storm during COVID-19 disease, potentially predisposing these patients to a worse outcome.

Additionally, consistent with previous reports^9,28,29^, we observed a higher number of in-hospital deaths in patients with positive auto-Abs to type I IFNs compared to their counterparts (Supplementary Fig. S2). Although patients with and without auto-Abs to type I IFNs did not show overt clinical differences in COVID-19, they differed in plasma IL-17 levels (Table 1). An in vivo study demonstrated an inverse association between lung tissue levels of IL-17 and LAG3 cTregs/uTregs^20^, which might explain the lower frequency of LAG3 cTregs/uTregs in the blood of these patients. However, further research is needed to fully understand the mechanistic link between LAG3 expression and IL-17 regulation.

Furthermore, in this study, the blood level of IL-35^+^IL-10^+^ Breg correlated with COVID-19 disease severity. Previous reports showed an upregulation of IL-10 and EBi3 (IL-35) transcripts in Tregs of patients with severe COVID-19^15,24^. This could mostly be a counteract mechanism induced in response to immune hyperactivation. In a report of bacterial infection, IL-35-producing B cells were found to be upregulated and to increase susceptibility to infection via the suppression of innate immunity, lowering macrophage activation, and altering inflammatory T cells^7^. In another report of mycobacterial infection, elevation in the numbers of IL-35^+^IL-10^+^ B cells infiltrating into lung tissue was associated with the downregulation of effector Th1/Th17 cells and upregulation of Foxp3^+^ Tregs^8^. Accordingly, in our study, the blood level of IL-35^+^IL-10^+^ Bregs was associated with upregulation of Foxp3^+^ Tregs (cTregs) and downregulation of IL-17 and other proinflammatory cytokines, such as IL-6, TNFα, and IL-1β (Fig. 3, Supplementary Fig. S1). This suggests that a therapy that increases these subtypes during severe COVID-19 infection could result in better control of immunopathology of cytokine storm.

Interestingly tocilizumab and/or a high dose of VitD increased the blood levels of IL-35^+^IL-10^+^ Bregs and cTregs/uTregs subtypes and subsequently lowered blood levels of IL-6 and IL-1β during severe COVID-19 infection. Previously, tocilizumab and VitD were reported to increase the blood levels of Tregs and reduce proinflammatory cytokines in chronic inflammatory diseases such as rheumatoid arthritis^30,31^. The mechanism of how these medications enhance the frequency of these regulatory cells, however, requires further investigations.

### Study limitation

Our study has several limitations. Firstly, while we hypothesize that the observed increase in blood level of regulatory B and T cells, especially in the presence of auto-Abs to type I IFNs, resulted from an indirect natural immune response aimed at controlling excessive inflammation resulting from uncontrolled viral replication, the possibility of a direct effect of anti-IFN autoantibodies on these regulatory cells cannot be excluded. Therefore, it is essential to note that our study is limited by not investigating this potential link, highlighting the need for additional research to unravel such a mechanism. Secondly, although prior reports have indicated potential immunosuppressive roles of these regulatory cells in the context of COVID-19 inflammation and cytokine storms^15,24,32^, our study did not confirm the immunosuppressive effect of these regulatory cells. However, the increase in IL-10 and IL-35 in these regulatory subsets might be suggestive of their immunosuppressive potential. Additionally, our study was a cross-sectional analysis of the levels of these immune regulatory subtypes at the peak of cytokine storm, as the blood was collected at one time from severe patients admitted to ICU. Conducting longitudinal studies to track changes in these cell subsets over time could substantially enhance our understanding of their immunomodulatory effects in COVID-19 and their potential contributions to the management and treatment of the disease.

In summary, we identified distinct Treg and Breg subtypes in COVID-19 patients, most notably IL-35^+^ and IL-10^+^ producing Bregs, which showed a correlation with disease severity and the presence of auto-Abs to type I IFNs.

### Supplementary Information

Supplementary Figure S1.

Supplementary Figure S2.

## Data Availability

Data used for this study is available upon reasonable request from the corresponding author.
