# Supplementary material for: Increased blood immune regulatory cells in severe COVID-19 with autoantibodies to type I interferons
Source: Sci Rep. 2023 Oct 13;13:17344. doi: 10.1038/s41598-023-43675-w (PMC10575900; doi:10.1038/s41598-023-43675-w)
Supplement: Supplementary file 1 — Supplementary Figure S1. [file 41598_2023_43675_MOESM1_ESM.pdf]

	cTreg	uTreg	LAG3 cTreg	LAG3 uTreg	IL-10 IL-35 Breg	CRP	D-Dimer	Ferritin	IL-6	IL-17	TNF	IL-1B
cTreg	1	.889**	-0.250	-0.228	.459**	-0.236	0.063	-0.060	-.290*	0.158	-0.109	-.282*
uTreg	.889**	1	-.396**	-.362*	.365*	-0.117	-0.052	0.024	-0.228	0.140	-0.026	-0.176
LAG3cTreg	-0.250	-.396**	1	.971**	0.104	-0.175	0.030	-0.117	0.091	-.285*	-.286*	-0.014
LAG3uTreg	-0.228	-.362*	.971**	1	0.128	-0.173	0.053	-0.130	0.112	-0.263	-.368*	-0.114
IL-10 IL-35 Breg	.459**	.365*	0.104	0.128	1	-.491**	-0.239	-.331*	-.353*	0.140	-.401**	-.462**
CRP	-0.236	-0.117	-0.175	-0.173	-.491**	1	.279*	.547**	0.171	0.046	.283*	0.142
D-Dimer	0.063	-0.052	0.030	0.053	-0.239	.279*	1	0.014	0.001	0.095	-0.114	-0.079
Ferritin	-0.060	0.024	-0.117	-0.130	-.331*	.547**	0.014	1	-0.098	-0.018	0.107	-0.046
IL-6	-.290*	-0.228	0.091	0.112	-.353*	0.171	0.001	-0.098	1	.294*	0.056	0.194
IL-17	0.158	0.140	-.285*	-0.263	0.140	0.046	0.095	-0.018	.294*	1	0.217	-0.116
TNF	-0.109	-0.026	-.286*	-.368*	-.401**	.283*	-0.114	0.107	0.056	0.217	1	.364*
IL-1B	-.282*	-0.176	-0.014	-0.114	-.462**	0.142	-0.079	-0.046	0.194	-0.116	.364*	1

Supplementary Figure S1. The correlation between levels of cTreg/uTreg, LAG3^{+} cTreg/uTreg, IL-35 and IL-10 producing CD138+ CD1d+ Bregs in patient's PBMCs and serum markers of COVID-19 severity such as CRP, D-Dimer, ferritin, and proinflammatory cytokines such as IL-6, IL-17, TNFα, and IL-1β. Statistical test: Pearson's correlation or Spearman's coefficient was used based on the normality of data, with a two-sided test for significance ($P < 0.05$, considered significant).
