# Supplementary material for: Increased blood immune regulatory cells in severe COVID-19 with autoantibodies to type I interferons
Source: Sci Rep. 2023 Oct 13;13:17344. doi: 10.1038/s41598-023-43675-w (PMC10575900; doi:10.1038/s41598-023-43675-w)
Supplement: Supplementary file 2 — Supplementary Figure S2. [file 41598_2023_43675_MOESM2_ESM.pdf]

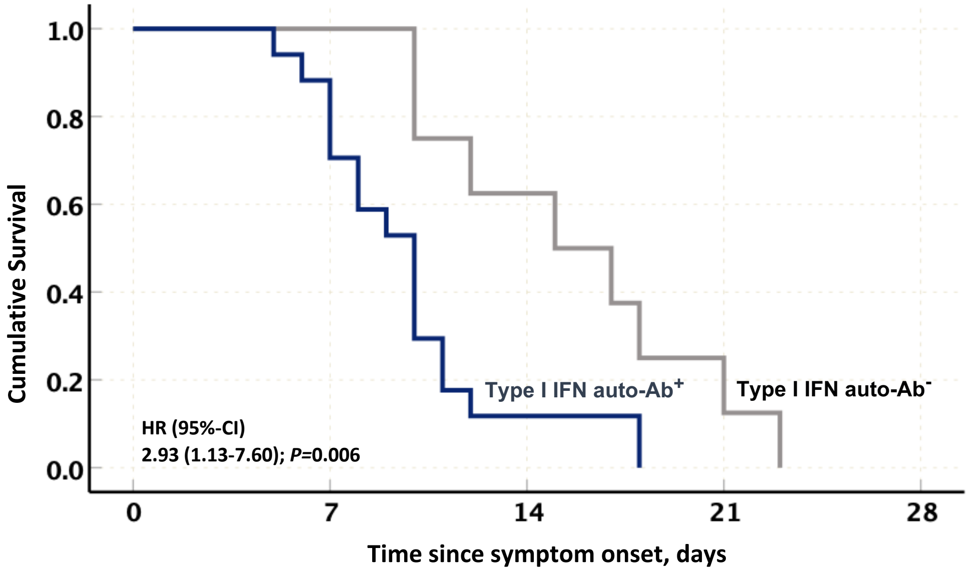

No. of death by 29 aays

auto-Abs IFNs⁻ (n=8)	0	0	4	3	1
auto-Abs IFNs⁺ (n=17)	0	5	10	2	0

Supplementary Figure S2. Higher 29-day all-cause mortality of patients with COVID-19 and auto-Abs to type I IFNs.
The Kaplan-Meier curve of patients with COVID-19 and auto-Abs to type I IFNs (n=18, 17 death events) or without auto-Abs to type I IFNs (n=20, 8 death events). The Cox proportional hazards regression model was adjusted for patient's demographics factors (age, gender, and body mass index), comorbidities (diabetes mellitus), and COVID-19 related severity serum marker (D-dimer and C-reactive protein).
